# Transitioning regulatory authorities in South Africa: a comparative review of the organisational structure and management practices

**DOI:** 10.1080/20523211.2026.2673693

**Published:** 2026-06-03

**Authors:** Anri Hattingh, Jacques Joubert, Michelle Viljoen

**Affiliations:** School of Pharmacy, University of the Western Cape, Cape Town, South Africa

**Keywords:** Medicines control council, South African Health Products Regulatory Authority, MCC, SAHPRA, medicine approval, regulatory affairs

## Abstract

**Background:**

South Africa’s transition from the Medicines Control Council (MCC) to the South African Health Products Regulatory Authority (SAHPRA) in 2018 marked a critical shift in the regulatory landscape. The MCC faced prolonged timelines, inadequate resources and a growing application backlog. This review comparatively examines how organisational, governance, managerial and procedural reforms introduced under SAHPRA (2018–2022) addressed these structural and performance limitations, and assesses their implications for regulatory policy and future system strengthening.

**Method:**

A mixed-method literature review combining qualitative thematic synthesis and quantitative descriptive analysis was conducted, drawing on publicly available sources, including government reports, academic journal articles, and organisational websites. Quantitative data (e.g. median approval timelines, backlog volumes and approval numbers) were systematically extracted from included studies and official reports without primary statistical modelling. Keywords included ‘SAHPRA', ‘MCC', ‘management structures’, ‘organisational structures', ‘regulatory review process', ‘approval timelines', ‘medicine approval', and ‘regulatory authorities'. Screening followed predefined inclusion and exclusion criteria, and data extraction captured governance structures, funding models, quality systems, reliance pathways, review timelines and backlog metrics.

**Results:**

SAHPRA’s implementation of reliance-based review pathways and risk-based assessments, alongside streamlined operations, reduced median approval times from 2,124 in 2018 to 783 days in 2020, and increased new chemical entity registrations from 15 to 155 over the same period. The inherited backlog (8,220 new applications in 2018) was cleared by 2022. The transition improved financial independence, enhanced institutional autonomy and reduced administrative bottlenecks. SAHPRA also demonstrated responsiveness during the COVID-19 pandemic through expedited review pathways.

**Conclusion:**

The transition from MCC to SAHPRA represents a structural and procedural modernisation of South Africa’s regulatory framework. While improving efficiency and access to essential medicines, findings should be interpreted cautiously due to reliance on secondary data and variation across sources. Continued digital reform, capacity development and strengthening post-marketing surveillance remain key priorities.

## Background

Rapid pharmaceutical innovation and market expansion have increased regulatory complexity, particularly in resource-constrained settings. Limited funding, outdated systems and technology, infrastructure challenges and restrained international and regional harmonisation hinder effective regulatory oversight, increasing the risk of substandard and falsified medicines. Strengthening medicine regulation is essential for protecting the public as regulatory authorities provide oversight and accountability throughout the pharmaceutical lifecycle to enhance the overall benefit-risk profile of medicines. Robust regulatory frameworks ensure that medicines remain safe, effective and of high quality once introduced into the market (Garashi et al., 2022; Ndagije et al., 2023; Vaishnavi, 2024). Establishing and maintaining robust regulatory systems requires skilled personnel and sustained investment in inspections and post-marketing surveillance (WHO, 2021a). As a result, many low-and middle-income countries struggle to keep pace with scientific advances. This may leave patients vulnerable with delayed healthcare access which is a basic human right or the potential to exposure of substandard and falsified medical products (Danks et al., 2023; Leng et al., 2015; Roth et al., 2018).

South Africa’s formal regulatory framework began with the Medicines and Related Substances Act, 1965 (Act 101 of 1965), and the Medicines Control Council (MCC) oversaw medicine approvals and clinical trials, among other functions (Keyter, Banoo, et al., 2018; Republic of South Africa, 2017). During the 1990s and early 2000s, subsequent amendments and policy updates sought to broaden the MCC’s mandate, this included the initial regulation of medical devices, introducing streamlined procedures and early digital management initiatives; however, these changes did not fully resolve underlying capacity and workflow challenges. Over subsequent decades, constrained resources, outdated workflows, and reliance on external reviewers led to prolonged approval times and mounting backlogs, underscoring the need for a more efficient, modernised authority (Keyter, Banoo, et al., 2018).

By 2005, the MCC was inundated with applications, more than doubling its prior annual submissions (Leng et al., 2015). This surge created a growing backlog and process bottlenecks in dossier evaluations. Complaints mounted that the authority’s slow turnaround time hindered access to affordable treatments, an especially pressing problem given South Africa’s significant burden of infectious and chronic diseases. As the agency struggled to cope, calls for legislative and structural reforms intensified (Keyter, Banoo, et al., 2018).

One pivotal moment was the Ministerial Task Team convened in 2006 under Professor Green-Thompson to investigate the MCC’s shortcomings. Their subsequent 2008 report highlighted the rising complexity of pharmaceutical products, the administrative inefficiencies of the MCC’s review processes, and the absence of harmonisation with other regulatory authorities. In response, they recommended the establishment of a new, more agile regulatory authority, capable of leveraging international best practices and streamlining the registration process. Over time, this recommendation led to the establishment of the South African Health Products Regulatory Authority (SAHPRA) (Green-Thompson, 2008; Keyter, Banoo, et al., 2018).

Legislative momentum for SAHPRA built steadily with the Medicines and Related Substances Amendment Act, 2008 (Act 72 of 2008) and the Amendment Act, 2015 (Act 14 of 2015) (Republic of South Africa, 2020). SAHPRA was formally launched as an autonomous Schedule 3A Public Entity during February 2018 (Dhiman & Dureja, 2021). Within the South African public finance framework, a Schedule 3A Public Entity operates outside direct departmental control, retains revenue generated through user fees, and is governed by an independent board accountable through the Public Finance Management Act. This designation therefore confers greater operational and financial autonomy than a statutory council embedded within a national department (Dhiman & Dureja, 2021; Keyter, Banoo, et al., 2018).

The newly established SAHPRA had to contend with fundamental barriers as South Africa’s pharmaceutical market continued to grow and new, more complex medical technologies require evaluators with specialised expertise and skills. Achieving a well-trained, stable workforce has been one of the authority’s greatest challenges. The backlog inherited from the MCC persisted and the urgent priority was on the lengthy evaluation time for dossier evaluations that delays life-saving treatments and undermines public confidence in the regulatory system (Keyter, Gouws, et al., 2018).

This review focused specifically on the evolution of medicine regulation in South Africa by examining the transition from the Medicines Control Council (MCC) to the South African Health Products Regulatory Authority (SAHPRA) during 2018–2022 after introducing organisational, governance and procedural reforms. The objectives were to comparatively evaluate (1) the organisational and governance structures of the MCC and SAHPRA; (2) managerial and review practices adopted before and after the transition; (3) performance indicators such as approval timelines, backlog reduction and number of applications received; (4) the MCC challenges in order to identify and assess if these implementations by SAHPRA strengthen regulatory policy and system strengthening.

## Methods

### Study design

This study employed a mixed-method comparative review design integrating qualitative thematic synthesis and quantitative descriptive extraction. Quantitative analysis was limited to extraction and comparison of already reported descriptive metrics (e.g. median overall regulatory approval time (in days), backlog volumes, number of applications approved per year and by review categories where available) from published studies and official reports. No independent primary datasets were analysed and no inferential statistical testing was performed.

### Search strategy

A comprehensive search was conducted across multiple electronic databases and search engines: EBSCOhost (multidisciplinary content), PubMed, ScienceDirect, and Scopus (peer-reviewed research), Google Scholar (grey literature), and SAePublications (South African regulatory publications) limited to English-language sources published between 2000 and 2024. Searches were supplemented by official guidelines and policy documents obtained from the SAHPRA website. Key terms such as ‘SAHPRA', ‘MCC', ‘organisational structures', ‘regulatory review process', ‘approval timelines', ‘medicine approval' and regulatory authorities were used individually and in combination, with Boolean operators refining the results. Materials focusing exclusively on other regulatory authorities, lacking empirical or qualitative analysis of MCC/SAHPRA operations, or presented as non-evidence-based commentaries were excluded.

### Screening process

The initial database search yielded records that were first screened by title and abstract for relevance to objectives of this comparative analysis. The full-text review was evaluated against predefined inclusion and exclusion criteria. Inclusion criteria required that publications: (1) were published in English between 2000 and 2024, (2) focused on the MCC or SAHPRA regulatory framework in South Africa, (3) described organisational, managerial, or procedural aspects of the review process, and (4) appeared in peer-reviewed journals, official reports, or recognised industry publications. Exclusion criteria discarded sources that: (1) addressed other national or regional regulatory authorities exclusively, (2) lacked empirical data or qualitative analysis, or (3) were non-English opinion pieces without referenced evidence. Reasons for exclusion were documented (e.g. non-South African focus, missing data and commentary without referenced evidence).

### Data extraction

The following variables were systematically extracted and formed the basis for structured comparison in the thematic tabulated results (Table 1): (1) legal status and governance model; (2) mandate scope; (3) board composition and executive structure; (4) funding and fee mechanism; (5) quality management systems; (6) document management systems; (7) regulatory review process; (8) timelines and backlog. Decisions were logged in a Microsoft Excel table, noting the source, inclusion / exclusion criteria and rationale (e.g. out-of-scope focus, missing data). Where possible, data were cross-verified across multiple sources to minimise bias. As this review only incorporated publicly available materials, no ethics approval was required.

**Table 1.** Comparison of MCC and SAHPRA – governance, organisational, management, and review processes.

Category	MCC	SAHPRA
**Legal Status and Governance Model**		
Date of Establishment	1965 (Keyter, Banoo, et al., 2018 and Keyter, Gouws, et al., 2018)	Legally established in 2017 (Keyter, Banoo, et al., 2018) and launched in 2018(Dhiman & Dureja, 2021)
Type of Entity/Legal Status	Statutory body under the National Department of Health (Keyter, Banoo, et al., 2018 and Keyter, Gouws, et al., 2018)	Schedule 3A Public Entity operating independently of the Department of Health with operational autonomy and accountability (Dhiman & Dureja, 2021; Keyter, Banoo, et al., 2018)
**Mandate**		
Mandate	Limited mandate - Primarily human & veterinary medicines (Keyter, Banoo, et al., 2018 and Keyter, Gouws, et al., 2018) - Act 101 of 1965 mentioned devices, but lacked formal regulations (Green-Thompson, 2008)	Extended mandate - Human & veterinary medicines, medical devices, complementary medicines, radiation-emitting products (Dhiman & Dureja, 2021; Keyter, Banoo, et al., 2018)
**Board composition and Executive Structure**		
Board Composition	Up to 24 members, including chairs of 11 expert committees (Keyter, Banoo, et al., 2018)	15 members, including chair, vice-chair, and experts from various fields (Dhiman & Dureja, 2021; Keyter, Banoo, et al., 2018)
Executive	Minister of Health, Director General of Health and Registrar as executive secretary (Keyter, Banoo, et al., 2018)	Led by a Chief Executive Officer, plus CFO, CRO, Company Secretary, Chief Manager (Dhiman & Dureja, 2021; Keyter, Banoo, et al., 2018)
Secretariat	Medicines Regulatory Affairs cluster with four directorates: (1) Operations & Administration (2) Inspectorate & Law Enforcement (3) Clinical Evaluation & Trials (4) Medicines Evaluation & Research (Green-Thompson, 2008; Keyter, Banoo, et al., 2018)	Five core programmes: (1) Administration (2) Authorisation Management (3) Inspectorate & Regulatory Compliance (4) Medicines Evaluation & Registration (5) Medical Devices, Diagnostics & Radiation Control (Dhiman & Dureja, 2021; Keyter, Banoo, et al., 2018)
Expert Committees	11 committees: Legal, CAMS, Names & Scheduling, Clinical Trials, Clinical, Pharmaceutical & Analytical, Biologicals, Veterinary, Medical Devices, Pharmacovigilance and GxP (Green-Thompson, 2008)	11 committees: Legal, CAMS, Names & Scheduling, Clinical Trials, Clinical, Pharmaceutical & Analytical, Biologicals, Veterinary, Medical Devices, Pharmacovigilance and GxP (Dhiman & Dureja, 2021; Keyter, Banoo, et al., 2018)
**Funding and Fee Mechanisms**		
Human Resources & Capacity	- Internal and external expertise to review applications - Relied heavily on external experts for reviews with lacking performances contracts and insufficient system to manage timeous reports (Keyter, Banoo, et al., 2018) - Inadequate in-house skill capacity (Leng et al., 2015) - Chronic shortages led to persisted backlogs (Dhiman & Dureja, 2021)	- Emphasis on internal capacity to reduce external reliance (Keyter, Banoo, et al., 2018) - Engaged local (15) & international (48) external evaluators to clear inherited backlog (Moeti et al., 2023b)
Fee Structure	- Revenue went to National Treasury - Lower fees (Green-Thompson, 2008; Keyter, Banoo, et al., 2018)	- Financially independent: can retain fees - Higher fee structure published in Gazette (Dhiman & Dureja, 2021; Republic of South Africa, 2020)
**Management Systems**		
Quality Management System (QMS)	None formalised (Keyter, Banoo, et al., 2018)	Dedicated QMS instituted, with external and internal audits (Keyter, Banoo, et al., 2018, 2021)
Continuous Improvement	- No routine external audits - No internal quality audits (Keyter et al., 2021)	- External audits scheduled - Internal audits *via* quality department - Backlog and business-as-usual tracking (Moeti et al., 2023a) - Expedient vaccine pathways during COVID-19 (Bhekisisa Centre for Health Journalism, 2022; Keyter et al., 2021)
**Document Management Systems**		
Document Management	Paper-based system (Keyter, Banoo, et al., 2018)	Electronic management (Dhiman & Dureja, 2021; Keyter, Banoo, et al., 2018)
**Regulatory Review Processes**		
Harmonisation Initiatives	- Limited reliance mechanisms - Participated in ZaZiBoNa but no broad legal framework for reliance (Keyter, Banoo, et al., 2018; Keyter, Gouws, et al., 2018)	- Legislative support for reliance implemented by South African Health Products Regulatory Authority (SAHPRA) (2019) - Three reliance pathways: abridged, verified, or mutual recognition (South African Health Products Regulatory Authority (SAHPRA), 2022a; SAHPRA, 2023) - Good review practices to identify strategies for better alignment within ZaZiBoNa initiative (Moeti et al., 2023a; Sithole et al., 2021); other NRAs (Danks et al., 2023) and with African Medicines Regulatory Harmonisation (Sithole et al., 2024) to align efficiency in reviews models
Regulatory Review Process	- Type III full assessment for all applications (Keyter, Gouws, et al., 2018) - The longest median calendar days of 2092 until approval over period 2011–2017 (Moeti et al., 2023b)	- Four pathways: full review, abridged, verified, or recognition (the last three are reliance-based assessment) (Danks et al., 2023; South African Health Products Regulatory Authority (SAHPRA), 2023a) - Implementation of RBA reduced median approval time to 511 calendar days (Moeti et al., 2023b)
**Timelines and Backlog**		
Target Timelines & Milestones	- No formal target timelines for new chemical entities - Fast-track (250 days) rarely met. (Figure 2) Keyter, Gouws, et al., 2018 - New chemical entity applications median approval time increased from 1175 days in 2015 to 1466 days in 2017 (Figure 2) Keyter, Gouws, et al., 2018 - Accumulated and persisted backlog applications of 8220 by 2018 (Moeti et al., 2023a)	- Distinct timelines for business-as-usual (BAU) initiated in 2019 (Moeti et al., 2023a) - Gradual reduction in median approval times over initial period to 155 days in 2022 for new chemical entities (Keyter et al., 2021) - Backlog clearance programme initiated in 2019 and it was cleared in 2022 (South African Health Products Regulatory Authority (SAHPRA), 2022b; Tomlinson, 2022)

*CAMS, Complementary and Alternative Medicines; GxP, Good Practices; NRAs, National Regulatory Authorities; QMS, Quality Management System; CEO, Chief Executive Officer; CFO, Chief Financial Officer; CRO, Chief Regulatory Officer; RBA, reliance-based assessment; ZaZiBoNa, collaborative initiative among Southern African Development Community Collaborative Medicines Registration Initiative.

### Comparative data analysis

Comparative analysis was conducted by organising the above extracted variables into thematic domains. These comparisons formed the basis for a narrative synthesis that contrasted MCC’s historical practices with SAHPRA’s reforms, assessing patterns, contrasts and reported outcomes across the two regulatory frameworks. This analytical framework guided both the ‘Results' and ‘Discussions' sections.

## Results

A total of 22 sources were finally included in this assessment of the transition of the MCC to SAHPRA. The majority (*n* = 7) were literature reviews, observational studies (*n* = 5), SAHPRA documents (*n* = 5), Government Gazettes (*n* = 2), newspaper articles (*n* = 2) and a government report (*n* = 1), listed in Supplemental Appendix A. These documents provided both qualitative insights into organisational change, management practices, and regulatory reforms and quantitative metrics such as cumulative backlogs and median approval time.

A comparative analysis was compiled to provide a comprehensive understanding and mapping of the most significant operational differences between the MCC and SAHPRA. Descriptive data extracted from the sources listed in Supplemental Appendix A was organised into a comparative table (Table 1). This comparative analysis evaluated and highlighted the differences and similarities between the MCC and SAHPRA across various thematic domains as reflected in Table 1.

### Legal and governance autonomy

A key structural distinction between the MCC and SAHPRA relates to legal status and governance autonomy. The MCC operated under the National Department of Health, whereas SAHPRA functions as a Schedule 3A Public Entity (Table 1). Establishing SAHPRA as a Schedule 3A public entity enabled it to retain and manage fees for organisational enhancements, overcoming a key barrier under the MCC. SAHPRA is a more autonomous and modern regulator. In addition, SAHPRA’s legislative positioning provides clearer governance structures, defined accountability lines, and greater operational independence. In contrast, the MCC’s integration within the Department of Health limited its flexibility in decision-making and responsiveness to emerging regulatory demands (Dhiman & Dureja, 2021; Keyter, Banoo, et al., 2018).

### Mandate

The new mandate of SAHPRA was broader than that of the MCC (Table 1), encompassing complementary medicines, medical devices, *in vitro* diagnostics, radiation-emitting products as well as the existing responsibilities for human and veterinary medicines (Dhiman & Dureja, 2021; Keyter, Banoo, et al., 2018). Unlike the MCC, which had been embedded within the National Department of Health’s administrative structure, SAHPRA was established with enhanced financial independence and operational independence, enabling it to fulfil this expanded mandate more effectively (Keyter, Banoo, et al., 2018).

### Board composition and executive structures

The SAHPRA board composition was streamlined from 24 to 15 members (Table 1), boosting accountability and decisiveness. The executive and secretariat were redesigned and overhauled in contrast to MCC’s limited statutory flexibility and fragmented structure. The four directorates were replaced with five core programmes (Table 1) responsible for conducting all the regulatory activities, while retaining the same number of expert committees (*n* = 11) to provide technical expertise and guidance (Dhiman & Dureja, 2021; Keyter, Banoo, et al., 2018). SAHPRA shifted towards a more centralised and professionalised operational model by strengthening its internal workforce. The MCC relied heavily on large committees and part-time external evaluators, which contributed to inefficiencies, inconsistent decision-making, and delays in processing complex applications. SAHPRA further prioritised the development of a permanent in-house review capacity, reducing reliance on external reviewers and improving continuity, accountability, and institutional memory. This restructuring has enabled clearer governance lines and improved coordination across regulatory functions

### Funding and fee mechanisms

SAHPRA as a decision-making authority is financially independent with control over its own budget and accountability mechanisms. This shift enabled it to set and retain its fees, thus potentially increasing resources for enhancing internal staffing, training (Table 1), and engaging with local and international evaluators to assist with the inherited backlog (Keyter, Banoo, et al., 2018; Moeti et al., 2023b). In contrast, the MCC’s financial model was closely tied to the National Treasury, limiting its ability to reinvest generated revenue into operational improvements. This dependency constrained investments in staff retention, digital infrastructure, and quality management systems.

### Management systems

SAHPRA’s conceptual, regulatory, management redesign including the overhauling of fee structures enabled SAHPRA to implement a dedicated quality management system (QMS) to institute audits (internal and external), to digitise workflows and responsiveness. Continuous quality improvement initiatives to reduce the backlog and expedite registrations were evident during COVID-19 vaccine registration pathways (Bhekisisa Centre for Health Journalism, 2022; Keyter et al., 2021; Keyter, Gouws, et al., 2018). A notable advancement was the implementation of the electronic document management system , which improved application tracking, transparency and process efficiency. In contrast, the MCC lacked formalised QMS, routine internal audits and comprehensive digital infrastructure. Its reliance on paper-based processes contributed to backlogs and limited its ability to monitor performance systematically (Dhiman & Dureja, 2021).

### Regulatory review process

MCC conducted Type III full assessments for the majority of applications regardless of prior international approval. With the introduction of reliance-based assessments (abridged, verification or mutual recognition) and harmonisation, SAHPRA enabled reliance on other regulatory authorities, thereby reducing duplication of scientific assessment and reallocated internal capacity towards higher-risk applications (Danks et al., 2023; Moeti et al., 2023b; Sithole et al., 2021, 2024). While MCC participated in limited collaborative initiatives such as ZaziBoNa (Keyter, Banoo, et al., 2018; Keyter, Gouws, et al., 2018; Sithole et al., 2021), these efforts were constrained by legislative and operational limitations. In contrast, SAHPRA implemented formally legislated reliance pathways, providing a structured and scalable approach to regulatory collaboration to expedite reviews (South African Health Products Regulatory Authority (SAHPRA), 2022a) for critical products, including vaccines, which facilitated improved patient accessibility as observed during public health emergencies such as COVID-19 (Bhekisisa Centre for Health Journalism, 2022; South African Health Products Regulatory Authority (SAHPRA), 2022a, 2023a; Keyter et al., 2021).

### Timelines and backlog

**Figure 1.**
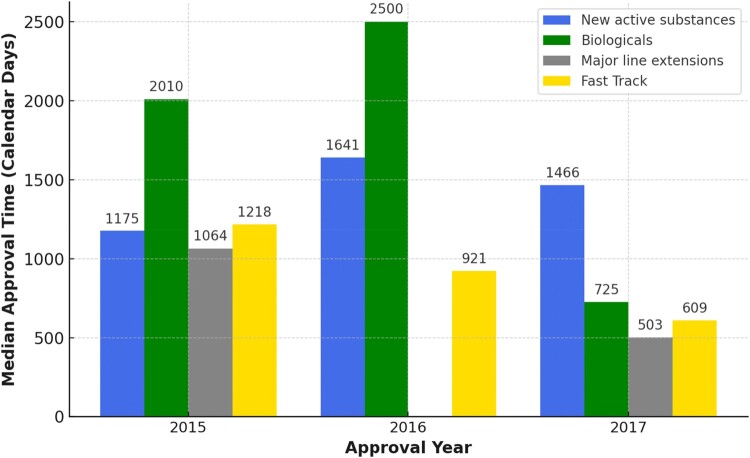
Median overall regulatory approval times (calendar days) for new active substances, biologicals major line extensions, and fast-track applications by MCC (2015–2017) (adapted from Keyter, Gouws, et al., 2018).

Both Table 1 and Figure 1 highlight how SAHPRA’s overhaul and interventions reduced the median overall regulatory approval time from 2 124 in 2018 to 783 days in 2020 and increased the number of new chemical entities from 15 in 2018 to 155 in 2020 (Keyter et al., 2021). The clearance of the systematic backlog was successfully completed by 2022 (South African Health Products Regulatory Authority (SAHPRA), 2022b; Tomlinson, 2022; Moeti et al., 2023a). In contrast, the MCC did not have formal target timelines for new chemical entities and rarely met the fast-track timeline of 250 days (Keyter, Gouws, et al., 2018).

This investigation additionally focused on the barriers, bottlenecks, and challenges faced by MCC. The main findings are summarised in Table 2, which depicts the crucial and some common overlapping barriers, bottlenecks, and challenges encountered by the MCC published from 2000 to 2024. The following identified recurring themes experienced by the MCC included: heavy reliance on part-time reviewers, outdated workflows, increased volumes of new applications and persistent backlogs, financial and resource constraints, absence of an electronic management system and legislative gaps (Dhiman & Dureja, 2021; Green-Thompson, 2008; Keyter et al., 2021, 2022; Keyter, Banoo, et al., 2018; Keyter, Gouws, et al., 2018; Keyter, Salek, Gouws, et al., 2019; Leng et al., 2015). These barriers and challenges are attributed to structural pitfalls such as extensive delays, high staff turnover, insufficient transparency and accountability and inconsistent decision-making that is clearly shown in Table 2.

**Table 2.** The barriers, bottlenecks, and challenges encountered by the MCC.

Author(s) & year	Barriers, bottlenecks, and challenges identified
Dhiman and Dureja (2021)	• Extensive timelines for approval of medicine registration applications • High application volumes (clinical trials, new chemical entities) • Lack of regulatory policies • Accumulating backlog • Overdependence on part-time external experts • Limited performance oversight by executives • Insufficient transparency and accountability • Inadequate financial autonomy • Absence of formal quality/document management system • Lack of harmonisation initiatives due to weak support structures
Green-Thompson (2008)	• Heavy reliance on a small pool of external reviewers with other primary jobs • No service-level agreements, so external reviewers did not adhere to predefined timelines • Lack of transparency and communication within the MCC • Minimal youth representation in MCC or among reviewers • High staff turnover • No electronic document management system • Poor coordination between directorates • Inadequate monitoring/tracking of applications • Ineffective reviewer task assignments • Low financial support; revenue went to Treasury • Limited financial resources • No clear strategy to develop regulatory expertise • Potential conflicts of interest with external reviewers • Lack of autonomy • Missed approval targets, resulting in prolonged timelines
Keyter, Banoo, et al. (2018)	• Rising volume of applications • Reliance on external reviewers for medicine registration • No dedicated quality or electronic document management system • Lack of financial support • Limited harmonisation practices in evaluation • Weak regulatory convergence and collaboration with other authorities
Keyter, Gouws, et al. (2018)	• Financial resource constraints • Over-reliance on external reviewers (no contractual time-bound obligations) • High volume of applications • Backlog of registration applications • No formal internal quality policies to ensure consistency or transparency • Failure to implement good review practices, SOPs, and assessment templates • Lack of a dedicated unit to evaluate review quality • Absence of an electronic document management system • No routine external or internal quality audits
Keyter, Salek, Gouws, et al. (2019)	• Resource constraints • Overwhelming volume of registration applications • Failure to define critical milestones in the review process • No specific target timelines for each milestone • Ongoing backlog of medicine registration
Keyter et al. (2021)	• Operational obstacles within the MCC’s organisational structure • Inadequate human and financial resources
Keyter et al. (2022)	• Large number of registration applications • Insufficient human and financial resources • Persistent backlog • Manual document management system • Legislative constraints preventing introduction of facilitated regulatory pathways
Leng et al. (2015)	• Lack of human resources • High staff turnover • Dependence on external reviewers for application evaluations • Inadequate organisational infrastructure • No electronic document tracking or robust regulatory procedures • MCC methods lagged behind modern pharmaceutical developments • Continuing backlog in medicine registration

The median overall regulatory approval timelines in calendar days for new active substances, biologicals, major line extensions and fast-track applications submitted to the MCC from 2015 to 2017 are illustrated in Figure 1. The longest median overall regulatory approval was 2,500 calendar days observed for biological applications submitted to the MCC during 2016 and it was reduced to 725 calendar days in 2017. The MCC was unable to achieve the target timeline of 250 calendar days that was set for the review of fast-track applications over the period 2015–2017 for any of the categories. Approval timelines from 2015 to 2017 misaligned with international standards, as the MCC had no formal targets, and the fast-track pathway never met its 250-day goal (Keyter, Gouws, et al., 2018).

**Figure 2.**
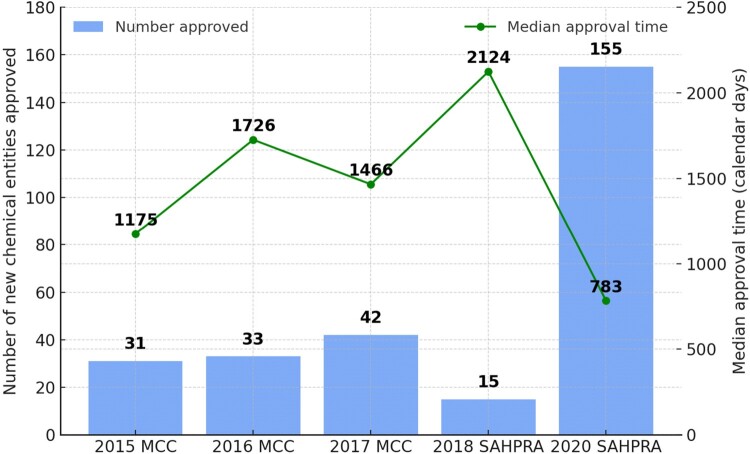
New chemical entities approved (blue) and median overall regulatory approval time in days (green) by MCC (2015–2017) and SAHPRA (2018 and 2020) (Keyter et al., 2021).

Figure 2 illustrates the number of new chemical entities approved, as well as the median overall regulatory approval times (calendar days) achieved by the MCC (2015–2017) and SAHPRA (2018 and in 2020). During the launching year (2018) of SAHPRA, they only approved 15 new chemical entities in a median approval time of 2,124 calendar days. This significantly improved to the highest approval number of 155 in the shortest time of 783 calendar days during 2020 compared 2015–2017. No data was publicly available for 2019 to explain or mitigate circumstances during this time period.

SAHPRA undertook a study to explore the implementation of a new review pathway termed the risk-based assessment (RBA). The study was conducted retrospectively on applications that were submitted to the MCC during 2006–2015 and were later integrated into the backlog project which was initiated by SAHPRA as the Backlog Clearance Project (BCP) in 2019. The backlog stream included all applications that the MCC received prior to the launch of SAHPRA in February 2018. The accumulative backlog inherited by SAHPRA for medicine registration applications in the pre-registration phase was 8220 (Keyter et al., 2021; Moeti et al., 2023a, 2023b) and approximately 16 000 applications which included variations by 2018 (South African Health Products Regulatory Authority (SAHPRA), 2022a, 2022b; Moeti et al., 2023b). Due to the implementation of the BCP by SAHPRA, they successfully cleared the inherited backlog by December 2022 (South African Health Products Regulatory Authority (SAHPRA), 2022b; Tomlinson, 2022).

## Discussion

This review study compared South Africa’s historical MCC and SAHPRA frameworks (2018–2022), to assess organisational restructuring, management reforms, and operational changes. The transition of MCC to SAHPRA introduced structural reforms designed to address longstanding governance, operational, financial, review processes and workflow inefficiencies. In reflecting on these findings, it is evident that SAHPRA’s establishment as an autonomous Schedule 3A public entity in 2018 introduced numerous benefits, including an extended mandate, more streamlined management processes, generation and retainment of revenue, extended reliance-based review pathways to reduce regulatory assessment times and optimise the registration processes. SAHPRA’s legislative positioning provides clearer governance structures, defined accountability lines, and greater operational independence. In contrast, the MCC’s integration within the Department of Health limited its flexibility in decision-making and responsiveness to emerging regulatory demands. This shift to an autonomous governance model aligns with international best practices, where regulatory authorities operate with defined independence while maintaining public accountability. SAHPRA is therefore positioned as a more autonomous and modern regulator (Dhiman & Dureja, 2021; Keyter, Banoo, et al., 2018).

While the MCC primarily focused on medicines, its ability to expand into additional product categories was constrained by limited statutory authority, despite attempts to oversee areas such as medical devices. In contrast, SAHPRA’s expanded legal remit reflects the increasing complexity and convergence of modern healthcare technologies, where distinctions between medicines, diagnostics, and devices are often blurred. Integrating these product categories under a single regulatory authority enables more coherent oversight, although it also increases the demand for technical expertise and resources (Dhiman & Dureja, 2021; Green-Thompson, 2008; Keyter, Banoo, et al., 2018).

The restructuring of SAHPRA with regards to operations, organisational structure and improvement of internal capacity has enabled clearer governance lines and improved coordination across regulatory functions (Dhiman & Dureja, 2021; Keyter, Banoo, et al., 2018; Leng et al., 2015).

SAHPRA’s cost-recovery model, although associated with increased regulatory fees, allows revenue to be reinvested directly into capacity building, information technology systems, and process optimisation. This approach reflects a more sustainable and globally aligned funding model, supporting long-term regulatory efficiency and resilience (Dhiman & Dureja, 2021; Keyter, Banoo, et al., 2018). This financial independence is consistent with best practices in many well-regarded regulators worldwide, such as in Australia, Canada, or parts of Europe, where cost recovery underpins robust oversight (Keyter et al., 2022).

A key aspect of SAHPRA’s regulatory reform was the adoption of reliance-based review models. In these pathways, also known as verification (Type I) or abridged review (Type II), SAHPRA accepts assessments already completed by WHO-listed reference National Regulatory Authorities (NRAs) (i.e. those benchmarked at maturity level 3 + or Pharmaceutical Inspection Co-operation Scheme members), thereby reducing redundant work (Keyter, Salek, Banoo, et al., 2019). Full reviews (Type III) are maintained for products that are new or lack prior approval from an accredited NRA. This tiered approach aims to ensure that critical resources are allocated intelligently. Rather than duplicating assessments on well-vetted products, SAHPRA can channel its limited manpower into novel treatments or higher-risk applications (South African Health Products Regulatory Authority (SAHPRA), 2022a; World Health Organization (WHO), 2021b).

Further supporting the reliance-based review models is ‘good review practices' (GRPs), which promote harmonisation, transparent decision-making, predictable timelines (World Health Organization (WHO), 2015) and expedite access to safe, effective and stable medicines (Danks et al., 2023; Sithole et al., 2021). The GRPs encourage the creation of consistent templates and standard operating procedures, which in turn enhance predictability for industry stakeholders and reduce avoidable errors or omissions. To complement GRPs, SAHPRA has committed to principles of ‘quality decision-making'. These frameworks revolve around consistent criteria, adequate risk-benefit assessments, and clearly recorded rationale for each decision, all of which help prevent biases or inconsistencies (Danks et al., 2023; Donelan et al., 2015). These developments reflect a shift towards risk-based, adaptive regulatory practices aligned with global harmonisation (Sithole et al., 2024) efforts.

The reorganisation and key reforms introduced by SAHPRA addressed many of the barriers, bottlenecks and challenges entrenched in the MCC inefficiencies. This is collectively demonstrated by the positive trajectory prompted by the transformed governance model, fee-based funding approach, and advanced regulatory approach adopted by SAHPRA. Although SAHPRA’s inaugural year (2018) still reflected extended timelines, the adoption of formalised reliance pathways, risk-based reviews, continuous improvements, overhauling the fee structure to secure reliable internal funding, digitising workflows, implementing digital tools and expanding in-house capacity yielded measurable gains. The implementation of a dedicated quality management system and the autonomy to hire trained staff for ongoing reviews and future initiatives (Dhiman & Dureja, 2021; Keyter, Salek, Gouws, et al., 2019) was associated with measurable improvements in reported efficiency and backlog reduction. The clearance backlog programme (CBP) eradicated the MCC’s inherited accumulated backlog of applications by the end of 2022 (South African Health Products Regulatory Authority (SAHPRA), 2021, 2022b; Tomlinson, 2022).

All these initiatives and reforms also shortened approval timelines across the board. Whereas the MCC’s median review times often exceeded 2,000 days, and even fast-track applications routinely missed the 250-day target (Green-Thompson, 2008; Keyter, Gouws, et al., 2018), SAHPRA’s streamlined practices cut median approvals to just a few hundred days for critical categories by 2020 (Keyter et al., 2021; Moeti et al., 2023a). A clear indicator of these reforms’ practical impact is how actual review times now align with established target timelines. For example, by 2020 SAHPRA achieved median overall regulatory approval times of approximately 783 days for new chemical entities and consistently met its 250-day fast-track target. SAHPRA, by contrast, has developed a more nuanced system distinguishing between business-as-usual and backlog process streams, with differential targets for new active substances and generic products (Keyter, Gouws, et al., 2018; Moeti et al., 2023a). Although initially some steps carried longer target times (e.g. the extended validation period), SAHPRA has generally instituted lower sponsor response times and robust reliance initiatives. The net result has been an overall tightening of approval windows, especially visible by 2020 in data showing median approval times significantly below those inherited from the MCC era (Keyter et al., 2021).

The transition to a new fee-based funding approach, digital tracking, introduction of reliance-based reviews, and a formalised quality management system represent structural reforms, while strengthened staffing and optimised ‘business as usual' processes coincided with improved regulatory turnaround indicators and decreased median approval times (South African Health Products Regulatory Authority (SAHPRA), 2022b; Moeti et al., 2023a). The consistent emphasis on building internal capacity rather than relying almost exclusively on external reviewers further contributes to SAHPRA’s operational stability. This expanded internal resource base also allows for more specialised review pathways such as risk-based or pilot studies aimed at optimising the timeline for specific products (Bhekisisa Centre for Health Journalism, 2022; Moeti et al., 2023a).

As SAHPRA matures into a stable, internationally recognised regulatory authority, it continues to face structural and operational challenges typical of regulators transitioning from legacy systems. A key limitation remains the alignment of its expanded mandate with available human and technical capacity. Although the shift to an in-house evaluator model has improved consistency and accountability, gaps persist in specialised areas such as biologics and advanced therapies, highlighting the need for sustained investment in skills development, retention, and institutional capacity (Keyter et al., 2021; Keyter, Banoo, et al., 2018).

Digital transformation represents another ongoing challenge. While SAHPRA has introduced electronic systems and digitised workflows, these remain in development, with hybrid processes and integration issues still affecting efficiency. Unlike the MCC’s paper-based limitations, SAHPRA must ensure continuous system optimisation, stakeholder training, and interoperability to sustain performance gains (Keyter, Salek, Gouws, et al., 2019; Moeti et al., 2023b).

Despite SAHPRA’s improved financial sustainability through strengthened financial independence and fee retention, it still requires careful management to balance cost recovery with equitable access. Over-reliance on industry fees may pose risks to market entry and regulatory independence, necessitating transparent and balanced fee structures aligned with public health priorities (Keyter, Banoo, et al., 2018; Dhiman & Dureja, 2021; World Health Organization (WHO), 2021b).

Similarly, while reliance-based review pathways have improved timelines, their long-term success depends on maintaining robust local benefit-risk contextualisation. Over-reliance on foreign regulatory decisions without sufficient local adaptation could undermine regulatory sovereignty (Keyter, Gouws, et al., 2018; South African Health Products Regulatory Authority (SAHPRA), 2022a).

Although SAHPRA has successfully reduced backlogs and improved timelines, sustaining these gains requires continuous workflow optimisation, proactive pipeline management, and real-time monitoring to prevent regression (South African Health Products Regulatory Authority (SAHPRA), 2022b; Moeti et al., 2023b).

Looking ahead, SAHPRA’s global competitiveness will depend on advancing regulatory maturity to be aligned with the WHO Global Benchmarking Tool indicators. SAHPRA needs to continue strengthening international collaboration, expanding participation in harmonisation initiatives such as ZaZiBoNa and broader national regulatory authority cooperation in Africa and globally (World Health Organization (WHO), 2021b; Ndomondo-Sigonda et al., 2021). In parallel, strengthening pharmacovigilance systems and post-market surveillance remains critical to ensure patient safety alongside accelerated approvals (Keyter et al., 2021). Sustained human capital development, supported by training pipelines and strategic partnerships, will underpin long-term regulatory resilience (Dhiman & Dureja, 2021; Keyter, Banoo, et al., 2018).

Ultimately, maintaining transparency, stakeholder engagement, and institutional independence will be essential for SAHPRA to consolidate its gains and remain a credible, efficient, and globally aligned regulatory authority (Ndomondo-Sigonda et al., 2021).

## Limitations of this review

The researchers did not have direct access to data from MCC or SAHPRA and were reliant on information published in the public sphere. This retrospective review study focused solely on the registration process of medicinal products and did not examine other aspects of the regulatory bodies, such as the roles and responsibilities of SAHPRA or the MCC with regard to clinical trials.

There were some discrepancies observed with median approval times and number of applications published for the registration of medicinal products. Green-Thompson (2008) and Moeti et al. (2023a) indicated contradictory findings regarding the number of approved applications and the median approval timeframes for 2006 and 2007. In addition, the analysis of the findings from Keyter, Gouws, et al. (2018) and Keyter, Salek, Gouws, et al. (2019) revealed minor discrepancies in the reported number of local and international applications approved in 2017 and the median approval timelines for new active substances, new chemical entities, biologicals, and major line extensions observed. These discrepancies underscore the limitations of relying exclusively on secondary published data. Variations in reported timelines, definitions of milestones, and categorisation of product types limit direct comparability across sources. It was not possible to differentiate all the indicators by review type and submission categories due to their retrospective secondary sourced nature. While secondary data suggest shortened review times and improved regulatory efficiency, conclusions regarding the direct causation should be interpreted cautiously.

## Conclusion

This study evaluated the transition and transformation of the initial South Africa’s medicine regulation agency known as MCC to the South African Health Products Regulatory Authority (SAHPRA) in 2018. The findings indicate that SAHPRA’s establishment represented a structural and organisational shift in South Africa’s medicine regulatory system and brought about major reforms and improvements, including enhanced autonomy, financial independence, organisational restructuring, streamlined operational processes, and reliance-based review pathways. These reforms were associated with improvements in approval times, backlog reduction and governance autonomy.

The transition from the MCC to SAHPRA was not without challenges. The MCC left a significant backlog of pending applications, and SAHPRA also faced financial and human resource constraints. Nevertheless, SAHPRA’s swift responses, demonstrated during the COVID-19 pandemic, highlight the authority’s ability to adapt to changing and emergency regulatory needs. The adoption of strategic, risk-based and reliance-driven processes signifies a modern, proactive approach to medicine regulation that aligns with global best practices, exemplified by the United States Food and Drug Administration breakthrough therapy designation and the European Medicine Agency adaptive pathways guidelines. SAHPRA’s recognition as a functional level of Maturity (Level 3 out of 4) by the World Health Organisation for national regulatory authorities further underscores its progress in delivering a stable, well-functioning and integrated regulatory system that ensures the quality, safety and efficacy of vaccines registered by SAHPRA (South African Health Products Regulatory Authority (SAHPRA), 2023b). In doing so, it has enhanced public access to affordable and essential medicines. This review also underscores the importance of robust resource management, quality decision-making and transparency for regulatory authorities.

The transition to SAHPRA offered policy-related lessons regarding governance autonomy, reliance-based review model, quality management systems and sustainable financing mechanisms for strengthening regulatory science in middle-income settings.

## Supplementary Material

Supplemental Material - Appendix

## References

[CIT0001] Bhekisisa Centre for Health Journalism. (2022). *Three ways COVID sped up SA’s medicine approvals*. Financial Mail. https://www.financialmail.businessday.co.za/fm/opinion/2022-11-07-3-ways-covid-sped-up-sas-medicine-approvals-process-and-how-it-can-help-the-nhi/.

[CIT0002] Danks, L., Semete-Makokotlela, B., Otwombe, K., Parag, Y., Walker, S., & Salek, S. (2023). Evaluation of the impact of reliance on the regulatory performance in the South African Health Products Regulatory Authority: Implications for African regulatory authorities. *Frontiers in Medicine*, *10*, 1265058. 10.3389/fmed.2023.1265058 37937144 PMC10626996

[CIT0003] Dhiman, S. K., & Dureja, H. (2021). SAHPRA – Relevance of the new South African Health Product Regulatory Authority and opportunity ahead. *Journal of Regulatory Science*, *9*(2), 1–14.

[CIT0004] Donelan, R., Walker, S., & Salek, S. (2015). Factors influencing quality decision-making: Regulatory and pharmaceutical industry perspectives. *Pharmacoepidemiology and Drug Safety*, *24*(3), 319–328. 10.1002/pds.3752 25628072

[CIT0005] Garashi, H. Y., Steinke, D. T., & Schafheutle, E. I. (2022). A systematic review of pharmacovigilance systems in developing countries using the WHO pharmacovigilance indicators. *Therapeutic Innovation & Regulatory Science*, *56*(5), 717–743. 10.1007/s43441-022-00415-y 35657484 PMC9356965

[CIT0006] Green-Thompson, R. W. (2008). *Report of the ministerial task team on the restructuring of the medicines regulatory affairs and medicines control council and recommendations for the new regulatory authority for health products*. Government of South Africa.

[CIT0007] Keyter, A., Banoo, S., Salek, S., & Walker, S. (2018). The South African regulatory system: Past, present and future. *Frontiers in Pharmacology*, *9*, 1407. 10.3389/fphar.2018.01407 30618735 PMC6300068

[CIT0008] Keyter, A., Gouws, J., Salek, S., & Walker, S. (2018). The regulatory review process in South Africa: Challenges and opportunities for a new improved system. *Therapeutic Innovation & Regulatory Science*, *52*(4), 449–458. 10.1177/2168479018776649 29848046 PMC6047299

[CIT0009] Keyter, A., Salek, S., Banoo, S., & Walker, S. (2019). The South African Medicines Control Council: Comparison of its registration process with Australia, Canada, Singapore, and Switzerland. *Frontiers in Pharmacology*, *10*, 228. 10.3389/fphar.2019.00228 30923501 PMC6426768

[CIT0010] Keyter, A., Salek, S., Banoo, S., & Walker, S. (2022). A proposed regulatory review model to support the South African health products regulatory authority to become a more efficient and effective agency. *International Journal of Health Policy and Management*, *11*(6), 795–809. 10.34172/ijhpm.2020.213 33300773 PMC9309913

[CIT0011] Keyter, A., Salek, S., Danks, L., Nkambule, P., Semete-Makokotlela, B., & Walker, S. (2021). South African regulatory authority: The impact of reliance on the review process leading to improved patient access. *Frontiers in Pharmacology*, *12*, 699063. 10.3389/fphar.2021.699063 34366850 PMC8342884

[CIT0012] Keyter, A., Salek, S., Gouws, J., Banoo, S., & Walker, S. (2019). Evaluation of the performance of the South Africa Regulatory Agency: Recommendations for improved patients’ access to medicines. *Therapeutic Innovation & Regulatory Science*, *54*(4), 878–887. 10.1007/s43441-019-00013-5 32557310 PMC7362885

[CIT0013] Leng, H. M. J., Sanders, D., & Pollock, A. (2015). Pro-generics policies and the backlog in medicines registration in South Africa: Implications for access to essential and affordable medicines. *Generics and Biosimilars Initiative Journal*, *4*(2), 58–63. 10.5639/gabij.2015.0402.014

[CIT0014] Moeti, L., Litedu, M., & Joubert, J. (2023a). The implementation of a risk-based assessment approach by the South African Health Products Regulatory Authority (SAHPRA). *Pharmaceutical Medicine*, *37*(1), 71–91. 10.1007/s40290-022-00452-w 36598646 PMC9877048

[CIT0015] Moeti, L., Litedu, M., & Joubert, J. (2023b). Regulatory registration timelines of generic medicines in South Africa: Assessment of the performance of SAHPRA between 2011 and 2022. *Journal of Pharmaceutical Policy and Practice*, *16*(1), 34. 10.1186/s40545-023-00537-0 36864490 PMC9983237

[CIT0016] Ndagije, H. B., Walusimbi, D., Atuhaire, J., & Ampaire, S. (2023). Drug safety in Africa: A review of systems and resources for pharmacovigilance. *Expert Opinion on Drug Safety*, *22*(10), 891–895. 10.1080/14740338.2023.2251375 37676033

[CIT0017] Ndomondo-Sigonda, M., Miot, J., Naidoo, S., Nelson, E. M., Ng’andu, B., Ngum, N., & Kaale, E. (2021). Harmonization of medical products regulation: A key factor for improving regulatory capacity in the East African Community. *BMC Public Health*, *21*(1), 187. 10.1186/s12889-021-10169-1 33478421 PMC7818747

[CIT0018] Republic of South Africa. (2017). *Medicines and Related Substances Act*, 1965 (Act 101 of 1965). *Government Gazette*, *40869*.

[CIT0019] Republic of South Africa. (2020). *Medicines and Related Substances Act, 1965 (*Act 101 of 1965 *as amended)*. *Government Gazette*, *44026*.

[CIT0020] Roth, L., Bempong, D., Babigumira, J. B., Banoo, S., Cooke, E., Jeffreys, D., Kasonde, L., Leufkens, H. G. M., Lim, J. C. W., Lumpkin, M., Mahlangu, G., Peeling, R. W., Rees, H., Ndomondo-Sigonda, M., Stergachis, A., Ward, M., & Nwokike, J. (2018). Expanding global access to essential medicines: Investment priorities for sustainably strengthening medical product regulatory systems. *Globalization and Health*, *14*(1), 102. 10.1186/s12992-018-0421-2 30382856 PMC6211488

[CIT0021] Sithole, T., Mahlangu, G., Capote, V., Sitoie, T., Shifotoka, S., Gaeseb, J., Padayachee, S., Sehloho, T., Khea, A., Fimbo, A., Munkombwe, Z., Mwale, B., Salek, S., & Walker, S. (2021). Evaluation of the good review practices of countries participating in the Southern African Development Community: Alignment and strategies for moving forward. *Frontiers in Medicine*, *8*, 742181. 10.3389/fmed.2021.742181 34513893 PMC8429788

[CIT0022] Sithole, T., Ngum, N., Owusu-Asante, M., Walker, S., & Salek, S. (2024). Comparison of three regional medicines regulatory harmonisation initiatives in Africa: Opportunities for improvement and alignment. *International Journal of Health Policy and Management*, *13*(1), 1–11. 10.34172/ijhpm.2024.8070 PMC11270597 39099506

[CIT0023] South African Health Products Regulatory Authority (SAHPRA). (2019). *Clinical guideline*. https://www.sahpra.org.za/wp-content/uploads/2020/02/2.09_Clinical-Guideline_Jul19_v2-1.pdf.

[CIT0024] South African Health Products Regulatory Authority (SAHPRA). (2021). *Backlog clearance programme – Extension of project*. https://www.sahpra.org.za/wp-content/uploads/2021/06/Backlog-Clearance-Programme_Extension-of-Project_22.06.2021_vF.docx.pdf.

[CIT0025] South African Health Products Regulatory Authority (SAHPRA). (2022a). *Reliance guideline (Doc. 5.08)*. https://www.sahpra.org.za/wp-content/uploads/2022/03/Reliance-Guideline_v3_22-Mar-2022.pdf.

[CIT0026] South African Health Products Regulatory Authority (SAHPRA). (2022b). *SAHPRA celebrates the conclusion of the backlog clearance project*. https://www.sahpra.org.za/wp-content/uploads/2022/12/MEDIA-RELEASE-Backlog-Clearance-02-December-2022.pdf.

[CIT0027] South African Health Products Regulatory Authority (SAHPRA). (2023a). *General information guideline (Doc.SAHPGL-HPA-07)*. https://www.sahpra.org.za/wp-content/uploads/2023/04/SAHPGL-HPA-07_v11-General-Information-Guideline.pdf.

[CIT0028] South African Health Products Regulatory Authority (SAHPRA). (2023b). *Annual performance plan 2023–2024*. https://www.sahpra.org.za/document/sahpra-annual-performance-plan-2023-2024/.

[CIT0029] Tomlinson, C. (2022). *How well did SA’s medicines regulator SAHPRA perform in 2022?* Daily Maverick. https://www.dailymaverick.co.za/article/2022-12-13-how-well-did-sas-medicines-regulator-sahpra-perform-in-2022/.

[CIT0030] Vaishnavi, A. (2024). Review on regulator’s perspective of TGA about pharmacovigilance during premarketing and post marketing of medicinal product. *Advances in Pharmacology & Clinical Trials*, *9*(3), 000249. 10.23880/apct-16000249

[CIT0031] World Health Organisation (WHO). (2015). Norms and standards: Good review practices. *WHO Drug Information*, *29*(1), 7–12.

[CIT0032] World Health Organisation (WHO). (2021a). *Good reliance practices in the regulation of medical products: High-level principles and considerations*. https://www.who.int/publications/m/item/annex-10-trs-1033.

[CIT0033] World Health Organisation (WHO). (2021b). *WHO global benchmarking tool (GBT): strengthening regulatory systems for medicines and vaccines*. https://www.who.int/publications/i/item/9789240020245.

