# Supplementary material for: Transitioning regulatory authorities in South Africa: a comparative review of the organisational structure and management practices
Source: J Pharm Policy Pract. 2026 Jun 3;19(1):2673693. doi: 10.1080/20523211.2026.2673693 (PMC13235249; doi:10.1080/20523211.2026.2673693)
Supplement: Supplemental Material - Appendix [file JPPP_A_2673693_SM3224.docx]

**Appendix A:** Summary of final sources included into comparison analysis (Supplementary)

|  | **Reference** | **Title (Shortened)** | **Source Type / Method** | **Key Focus** |
| --- | --- | --- | --- | --- |
|  | Danks et al., 2023 | Impact of reliance on the regulatory performance of SAHPRA | Journal Article | Use of reliance-based regulatory review to expedite registration of safe, effective and needed medications. |
| 2. | Department of Health (South Africa) (2017) | *Medicines and Related Substances Act, 1965 – Gov. Gazette 40869* | Government Gazette | Legislative framework for medicines regulation in South Africa |
| 3. | Department of Health (South Africa) (2020) | *Medicines and Related Substances Act, 1965 – Gov. Gazette 44026* | Government Gazette | Updates on fees and procedures for medicines regulation |
| 4. | Dhiman, S.K. & Dureja, H. (2021) | *SAHPRA – Relevance of the New South African Health Product Regulatory Authority and Opportunities Ahead* | Journal Article / Literature Review | SAHPRA’s roles, challenges, and collaborative potential |
| 5. | Green-Thompson, R.W. (2008) | *Report of the Ministerial Task Team on Restructuring the MCC & Recommendations for a New Authority* | Government Report | Proposed restructuring of MCC; basis for SAHPRA establishment |
| 6. | Keyter *et al*. (2018a) | *The South African Regulatory System: Past, Present, and Future* | Journal Article / Literature Review | Historical overview; evolution toward risk-based review in SAHPRA |
| 7. | Keyter *et al*. (2018b) | *The Regulatory Review Process in South Africa: Challenges and Opportunities* | Journal Article / Observational (Cross-Sectional) | Comparison of MCC vs. SAHPRA review practices; good review practices |
| 8. | Keyter *et al*. (2019b) | *Evaluation of the Performance of the South African Regulatory Agency* | Journal Article / Observational (Cross-Sectional) | Agency performance metrics; improving patient access |
| 9. | Keyter *et al*. (2021) | *South African Regulatory Authority: The Impact of Reliance* | Journal Article / Observational (Cross-Sectional) | Reliance pathways; backlog reduction; improved review times |
| 10. | Keyter *et al*. (2022) | *A Proposed Regulatory Review Model to Support SAHPRA* | Journal Article / Conceptual/Theoretical Review | Proposal for a more efficient review model (ZAPAR); alignment with global benchmarks |
| 11. | Leng *et al*. (2015) | *Pro-generics Policies and the Backlog in Medicines Registration in SA* | Journal Article / Literature Review | Registration backlog; pro-generics policies; impact on access to essential medicines |
| 12. | Moeti *et al*. (2023a) | *Implementation of a Risk-Based Assessment Approach by SAHPRA* | Journal Article / Observational | Introduction and outcomes of SAHPRA’s risk-based assessment |
| 13. | Moeti *et al*. (2023b) | *Regulatory Registration Timelines of Generic Medicines in SA (2011–2022)* | Journal Article / Observational | SAHPRA performance trends; timeline analysis for generic product approvals |
| 14. | Nkambule, P. (2022) | *Three Ways Covid Sped Up SA’s Medicine Approvals* | Online Newspaper Article / Spotlight | Covid-19’s impact on SAHPRA processes; National Health Insurance context |
| 15. | SAHPRA (2019) | *Clinical Guideline (Doc. SAHPGL-CEM-01)* | SAHPRA Guideline | Official guidance on clinical review processes |
| 16. | SAHPRA (2021) | *Backlog Clearance Programme – Extension of Project* | SAHPRA Document | SAHPRA’s strategic plan for clearing inherited backlog |
| 17. | SAHPRA (2022a) | *Reliance Guideline (Doc. 5.08)* | SAHPRA Guideline | Framework for reliance-based review pathways |
| 18. | SAHPRA (2022b) | *SAHPRA Celebrates Conclusion of the Backlog Clearance Project* | Media Release | Public announcement of backlog clearance success |
| 19. | SAHPRA (2023a) | *General Information Guideline (Doc. SAHPGL-HPA-07)* | SAHPRA Guideline | Broad administrative and technical requirements |
| 20. | Sithole et al., 2021 | *Evaluation of GRP Practices in the Southern African* | Journal Article | ZaZiBoNa initiative improved regulatory reviews in six countries. Other agencies in region and beyond to identify best practices. |
| 21. | Sithole et al., 2024 | *Harmonisation Initiatives in Africa* | Journal Article | African Medicines Regulatory Harmonisation can align efficiency in reviews models |
| 22. | Tomlinson, C. (2022) | *How Well Did SA’s Medicines Regulator SAHPRA Perform in 2022?* | Online Newspaper Article / Spotlight | SAHPRA performance review; WHO recognition for vaccine oversight |
|  |  |  |  |  |
